# In Vitro Effects of Twelve Food Additives on Gut Microbiome and Its Fibre Fermentation Capacity in Adults with Crohn’s Disease in Remission and Healthy Controls

**DOI:** 10.3390/nu18040668

**Published:** 2026-02-18

**Authors:** Hanoof Alessa, Molly Elizabeth Quinn, Linah Alhomidan, Cameron Ross, Stefanos Kainadas, Emily Brownson, Jonathan MacDonald, John Paul Seenan, Ben Nichols, Athanasios Koutsos, Konstantinos Gerasimidis

**Affiliations:** 1 Human Nutrition, School of Medicine, College of Medical, Veterinary and Life Sciences, University of Glasgow, New Lister Building, Glasgow Royal Infirmary, Glasgow G31 2ER, UK; 2421100a@student.gla.ac.uk (H.A.); 2710001q@student.gla.ac.uk (M.E.Q.); 2711664a@student.gla.ac.uk (L.A.); c.ross.3@research.gla.ac.uk (C.R.); stkainadas@gmail.com (S.K.); 2724161b@student.gla.ac.uk (E.B.); ben.nichols@glasgow.ac.uk (B.N.); athanasios.koutsos@glasgow.ac.uk (A.K.); 2 Department of Clinical Nutrition, College of Applied Medical Sciences, Imam Abdulrahman Bin Faisal University, Dammam 31441, P.O. Box 1982, Saudi Arabia; 3 Department of Gastroenterology, Queen Elizabeth University Hospital, Glasgow G51 4TF, UK; jonathan.macdonald2@nhs.scot (J.M.); johnpaul.seenan@ggc.scot.nhs.uk (J.P.S.)

**Keywords:** food additives, short-chain fatty acids, fibre, gut microbiome, Crohn’s disease

## Abstract

**Background/Objectives:** Animal studies have shown that food additives may adversely affect the gut microbiome. However, the effect of food additives on the microbiome in adults with Crohn’s disease (CD) remains less explored. This study investigated the impact of food additives on gut microbiome and fibre fermentation capacity in adults with CD and healthy controls (HCs) using in vitro faecal fermentations. **Methods:** Faeces from 6 HCs and 6 patients with CD in clinical remission (Harvey Bradshaw Index < 5) were used for in vitro fermentation of a fibre mix with one of 12 food additives (calcium propionate, carboxymethylcellulose, carrageenan kappa, cinnamaldehyde, maltodextrin, polysorbate-80, potassium sorbate, sodium benzoate, sodium sulphite, titanium dioxide, turmeric, and xanthan gum). Short-chain fatty acids (SCFAs) were measured using gas chromatography, the microbiome was profiled with 16S rRNA amplicon sequencing and total bacterial load was measured with qPCR. **Results**: Maltodextrin increased acetate production in both groups. In HCs, turmeric increased acetate and butyrate production, sodium sulphite reduced acetate production, and maltodextrin reduced butyrate production. Microbiome Shannon α-diversity increased with titanium dioxide (both groups), and with carrageenan kappa only in patients with CD. In both groups, the addition of maltodextrin and polysorbate-80 induced significant shifts in microbiome structure (β-diversity). Significant shifts were seen with maltodextrin (HC: R^2^ = 6.8%, *p* = 0.001; CD: R^2^ = 5.1%, *p* = 0.004) and sodium sulphite (HC: R^2^ = 6.9%, *p* = 0.001). Maltodextrin significantly decreased the estimated absolute abundance of *Escherichia–Shigella* in patients CD; sodium benzoate, potassium sorbate, and calcium propionate did so in HCs. *Faecalibacterium* decreased in the presence of polysorbate-80 in the HC and CD groups, as well as in the presence of maltodextrin in the CD group. Total bacterial load decreased with polysorbate-80, potassium sorbate, maltodextrin and calcium propionate in both groups. Xanthan gum decreased total bacterial load in HCs. **Conclusion:** Certain food additives significantly affected fibre fermentation capacity and microbiome structure, with only modest differences observed according to participants’ health status.

## 1. Introduction

Inflammatory bowel disease (IBD) is a chronic inflammatory condition of the gastrointestinal tract, characterised by episodes of relapse and remission [1]. Several studies have observed compositional and functional alterations in the gut microbiome of people with IBD, often associated with disease activity and phenotype [2]. Typical features of dysbiosis in Crohn’s disease (CD) include a decrease in beneficial organisms, such as *Faecalibacterium prausnitzii* and *Roseburia*, as well as an increase in potentially harmful organisms such as *Escherichia–Shigella* and *Enterococcus* [3]. Understanding the role of microbial changes in the disease course is of crucial importance in unravelling the aetiology of CD and improving disease management.

Diet is a major regulator of gut microbiome composition and function [4], with fibre fermentation by the gut microbiome having attracted the most interest. The metabolic end products of fibre fermentation, known as short-chain fatty acids (SCFAs), have significant beneficial effects on the host [5], which extend beyond the gut. As a prime example, butyrate modulates gene expression and intracellular signalling pathways and interacts with receptors on the cell surface, influencing host cell function [6]. Additionally, butyrate can reduce the production of proinflammatory cytokines and therefore has anti-inflammatory properties [7]. Notably, patients with IBD have reduced SCFA levels due to alterations in gut microbiome composition, leading to reduced fermentation of dietary fibre. A reduction in butyrate-producing bacteria may decrease levels of anti-NF-κB activity and increase the risk of inflammation in the gut [8].

Epidemiological studies and animal experiments have investigated how dietary patterns or specific food components influence the risk of IBD development or progression [9,10]. Recently, particular attention has been paid to food industrialisation and how food additives, abundantly used in the food chain, may mechanistically increase the risk of gut inflammation and by extension the risk of IBD onset. While food additives must be safe for consumption to be used in the food industry, their effect on the microbiome of patients with CD is largely unexplored [11]. In mice, acesulfame potassium, sucralose, sucrose, and aspartame have been shown to exert pro-inflammatory effects and reduce gut bacterial diversity [12]. Rinninella et al. [13] showed that consumption of titanium dioxide may aggravate gut barrier impairment and intensify immune responses in animals with colitis. A randomised double-blind controlled feeding study in healthy adults found that the participants who consumed a diet enriched with 15 g per day of carboxymethylcellulose for 11 days had reduced SCFA levels compared with those consuming an emulsifier-free diet [14]. Moreover, an in vitro study showed that sodium sulphite decreased acetate production, and cinnamaldehyde decreased butyrate production, compared with a no-additive control [15]. Likewise, De Souza Lopes et al. [16] observed in vitro antimicrobial effects of sodium benzoate and potassium sorbate against SCFA-producing bacteria, including *Lactococcus lactis* and *Lactobacillus acidophilus*.

While animal model studies provide valuable insights to unravel mechanisms of disease pathogenesis and are essential for drug development, there are significant limitations in their use as models of human IBD, including the fact that rodent or murine microbiomes are significantly different from the human one.

In vitro simulators of the human gut are powerful tools for studying the human gut microbiome due to their controlled experimental conditions, cost-effectiveness, high throughput and direct relevance to humans [17]. This study explored the impact of food additives on the gut microbiome of adults with CD in clinical remission and its fibre fermentation capacity and compared these effects against those observed in healthy controls (HCs) using in vitro batch faecal fermentations.

## 2. Materials and Methods

### 2.1. Participants

Faecal samples were collected from twelve adults (six patients with CD and six HCs) (mean [standard deviation (SD)]; age: 31.4 (11.7) years old; BMI: 24.4 (5.0) kg/m^2^). Participants with CD were in clinical remission (Harvey Bradshaw Index < 5), and none of the participants had received antibiotics in the two months prior to sample collection. A written consent form was obtained from all participants. The study was approved by the West of Scotland NHS GI Biorepository Committee.

### 2.2. In Vitro Batch Faecal Fermentation

Faecal samples were obtained immediately after defaecation and were kept on ice under anaerobic conditions (Thermo Scientific™, Oxoid™ AnaeroGen™ 3.5 L Sachet, Catalogue number: AN0035A, Manchester, UK) to reduce microbial metabolic activity. Samples were processed within two hours of defecation. A 16% (*w*/*v*) faecal slurry was prepared in sterile, oxygen-free Sorensen’s phosphate buffer, pH 7. Particulate matter from the faecal slurry was removed through straining using nylon stockings (30 denier). A volume of 5 mL of 16% faecal slurry was injected into a 100 mL fermentation glass vessel as well as a mixed fibre substrate (600 mg) consisting of α-cellulose (150 mg; SIGMA-Aldrich C8002, Gillingham, UK), high resistant maize starch (150 mg; National Starch^TM^, HI-MAIZE^TM^ 260, Manchester, UK), raftiline (150 mg; Beneo^TM^, Orafti P95, oligofructose produced by partial enzymatic hydrolysis from chicory inulin, Mannheim, Germany) and pectin from apple (150 mg; SIGMA-Aldrich 93854, Gillingham, UK). These fibres were chosen to best represent some of the most commonly consumed fibre in the UK, and the amounts were selected to reflect the recommended daily fibre intake of 30 g/day [18].

On the day of the experiment, fermentation medium was freshly prepared to a final volume of 1.5 L. The medium consisted of 675 mL 0.07 M tryptone solution, 337.5 mL buffer solution [NH_4_HCO_3_ (0.051 M), NaHCO_3_ (0.417 M)], 337.5 mL macromineral solution [Na_2_HPO_4_ (0.04 M), KH_2_PO_4_ (0.046 M), MgSO_4_.7H_2_O (0.002 M)], and 168.75 μL micromineral solution [CaCl_2_.2H_2_O (0.898 M), MnCl_2_.4H_2_O (0.505 M), CoCl_2_.6H_2_O (0.042 M), 1.6875 mL 0.1% resazurin solution]. The fermentation medium was adjusted to pH 7 with 6 M HCl, and the total volume was brought to 1.5 L with sterile distilled water. After boiling, degassing under oxygen-free nitrogen and cooling to 37 °C, porcine mucin (150 mg) and porcine bile acids (115 mg) were added. The fermentation medium was re-adjusted to pH 7 with 6 M HCl. The fermentation medium (42 mL) and 2 mL reducing solution [L-cysteine hydrochloride (0.039 M), NaOH (0.04 M), Na_2_S.9H_2_O (0.026 M)] were added to each fermentation bottle.

Thirteen fermentation bottles were prepared, including 12 food additives [potassium sorbate, calcium propionate, maltodextrin, carboxymethyl cellulose, polysorbate-80, carrageenan-kappa, titanium dioxide, sodium benzoate, sodium sulphite, cinnamaldehyde, turmeric, xanthan gum], and one no-food additive control, all incubated for 24 h. The amounts of food additives added were determined based on the Estimated Daily Intake (EDI) or the Acceptable Daily Intake for each additive, assuming that an average adult male weighs 75 kg (Table S1). For food additives with a high value of Estimated Daily Intake, the amount evaluated was standardised to 500 mg. For food additives with low daily consumption, the tested amount was set at 50% of the acceptable daily intake. The selection of these food additives was based on previous studies that reported their effects on intestinal inflammation and the pathogenesis of IBD [11,19].

From the no-food additive control (CNT) with fibre, one aliquot was collected immediately after faecal slurry inoculation at baseline (0hr-CNT), and a second aliquot was collected after 24 h of incubation (24hr-CNT). All other fermentation bottles were incubated for 24 h under oxygen-free nitrogen, and incubated in a water bath shaker at 37 °C at 60 strokes/min. Aliquots were collected for the determination of SCFA stocked in a 1:1 ratio with 1 M NaOH and stored at −20 °C until analysis. Aliquots collected for DNA extraction were stored in a −80 °C freezer.

### 2.3. SCFA Extraction and Analysis

The SCFAs (acetate, propionate, butyrate, valerate, caproate, caprylate and enanthate) and branched-chain fatty acids (BCFAs) (iso-butyrate and iso-valerate) were extracted from faecal slurries using diethyl ether as described previously [20,21]. Gas chromatography (7890A GC System, Agilent Technologies LDA, Cheadle, UK) with a flame ionisation detector was used for analysis of the SCFAs in the ether extracts. 2-Ethylbutyric acid (74.0 mM)] was used as internal standard. SCFA concentrations were quantified using standard curves derived from external standards of known SCFA concentrations [acetate (185.8 mM), propionate (144.5 mM], butyrate (114.2 mM), valerate acid (83.4 mM), caproate (52.6 mM), enanthate (65.8 mM), caprylate (53.2 mM), iso-butyrate (97.3 mM), and iso-valerate (87.0 mM)] that were added to 2 M NaOH with 2-ethylbutyric acid (74 mM) as the internal standard. The absolute concentration of SCFAs is reported (μmol) per volume (mL) of fermentation slurry.

### 2.4. Extraction of Genomic DNA from Fermentation Slurries

All the fermentation slurry samples (168 samples) were thawed at room temperature and then centrifuged at 15,000× *g* for 10 min. The supernatant was removed, and the genomic DNA from the pellets was extracted using the QIAGEN DNeasy Powersoil pro DNA Kit (QIAGEN, Hilden, Germany). Genomic DNA quality and quantity were evaluated using NanoDrop^TM^ 1000 (Thermo Fisher Scientific, Wilmington, Delaware, USA) and Qubit fluorometer (Thermo Fisher Scientific, Waltham, MA, USA). DNA extracts were sent to NU-OMICS for sequencing. Amplicon sequencing of the human gut microbiome was performed using the V4 region of the 16S rRNA gene, sequenced on an Illumina MiSeq platform (Illlumina, UK) following the Schloss 250 bp paired end method [22].

### 2.5. Quantification of Total Bacteria Load

The total bacterial load was measured using quantitative PCR (qPCR) (expressed as 16S ribosomal RNA gene copies per mL of fermentation slurry), using TaqManTM chemistry on a 7500 Real-Time PCR system (Applied Biosystems, Carlsbad, CA, USA), as described previously [20]. Serial dilutions of *Bacteroides vulgatus* were used as standards for absolute quantification. Each qPCR reaction (15 μL) consisted of 7.5 μL TaqManTM gene expression master mix, 2.25 μL nuclease-free water, 0.5 μL bovine serum albumin (20 mg/mL), 1.5 μL forward primer (9 µM), 1.5 μL reverse primer (9 µM) and 0.75 μL probe (2.5 µM). Each sample was analysed in duplicate. Total bacterial load was also used to estimate absolute abundances of bacterial groups using the relative abundances obtained from 16S rRNA amplicon sequencing.

### 2.6. Bioinformatics

Microbiome data were processed using the DADA2 pipeline version 1.16 [23]. An initial quality filtering step was carried out on raw sequencing data, eliminating reads with an expected error rate >2 and truncating those with an Illumina quality score <2. The DADA2 core algorithm was then utilised, employing machine learning to identify Amplicon Sequence Variants (ASVs) from paired-end reads. Subsequently, chimeras were removed via the DADA2 de novo chimera detection method. Taxonomic classification up to the genus level was assigned to each ASV using the RDP Naive Bayesian Classifier [24] algorithm alongside the SILVA 138 reference database [25] and ASVs matching exactly with the reference database were classified at the species level.

### 2.7. Microbiome and Statistics Analysis

Statistical analysis of microbiome data was carried out using R version 4.3.2. The dataset was analysed for α diversity using four metrics: Chao1 richness estimate, rarefied richness, Shannon diversity index, and Pielou’s evenness. Differences between groups were assessed using Mann–Whitney tests. The overall community composition was visualised using non-metric multidimensional scaling (NMDS) of Bray–Curtis dissimilarity matrices and evaluated through permutation ANOVA. Changes in ASV absolute abundances across time points were examined using paired Mann–Whitney tests.

Minitab Statistical Software (Minitab, 19.1.1) was used for statistical analysis of non-microbiome-based parameters. Normality of the data was assessed using the Anderson-Darling test. Differences in food additives between groups were explored using independent two-sample *t*-tests for normally distributed data and Mann–Whitney U tests for non-normally distributed data. Significance was determined as *p* < 0.05. Taxon abundances were expressed as absolute values using the qPCR estimate of total bacterial load multiplied by the relative abundance of each taxon.

## 3. Results

### 3.1. Effect of Food Additives on SCFA Production

**Figure 1:**
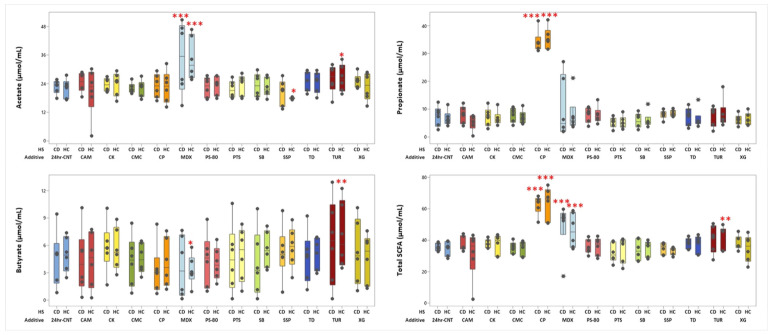
Net production of acetate, propionate, butyrate, and total short-chain fatty acids (SCFAs) after 24 h batch faecal fermentation of fibre with 12 different food additives and no food additive control in HCs (*n* = 6) and patients with CD (*n* = 6). Results are reported as μmol/mL faecal slurry. Abbreviations: 24hr-CNT, no-additive control; CP, calcium propionate; CMC, carboxymethylcellulose; CK, carrageenan kappa; CAM, cinnamaldehyde; MDX, maltodextrin; PS-80, polysorbate-80; PTS, potassium sorbate; SB, sodium benzoate; SSP, sodium sulphite; TD, titanium dioxide; TUR, turmeric; XG, xanthan gum; HS, health status; CD, Crohn’s diseases; HC, healthy control. Each dot represents an individual value. (* = *p* < 0.05, ** = *p* < 0.01, *** = *p* < 0.001 compared to no food additive control).

In both CD and HC groups, maltodextrin increased the in vitro production of acetate (*p* < 0.0001) compared with the no-additive control (24hr-CNT). In HCs, only turmeric increased the production of acetate (*p* = 0.032) and butyrate (*p* = 0.003), whereas sodium sulphite reduced acetate (*p* = 0.039) and maltodextrin reduced butyrate (*p* = 0.011) compared with the no-additive control (Figure 1). Notably, calcium propionate increased propionate production, but this was an artefact of the addition of calcium propionate in the fermentation reaction vessel. No other significant effects were observed for the remaining food additives. Other SCFAs (valerate, caproate, caprylate and enanthate) and branched-chain fatty acids (BCFAs) (iso-butyrate and iso-valerate) are displayed in Figures S1 and S2.

### 3.2. Effect of Food Additives on Microbiome Diversity

**Figure 2:**
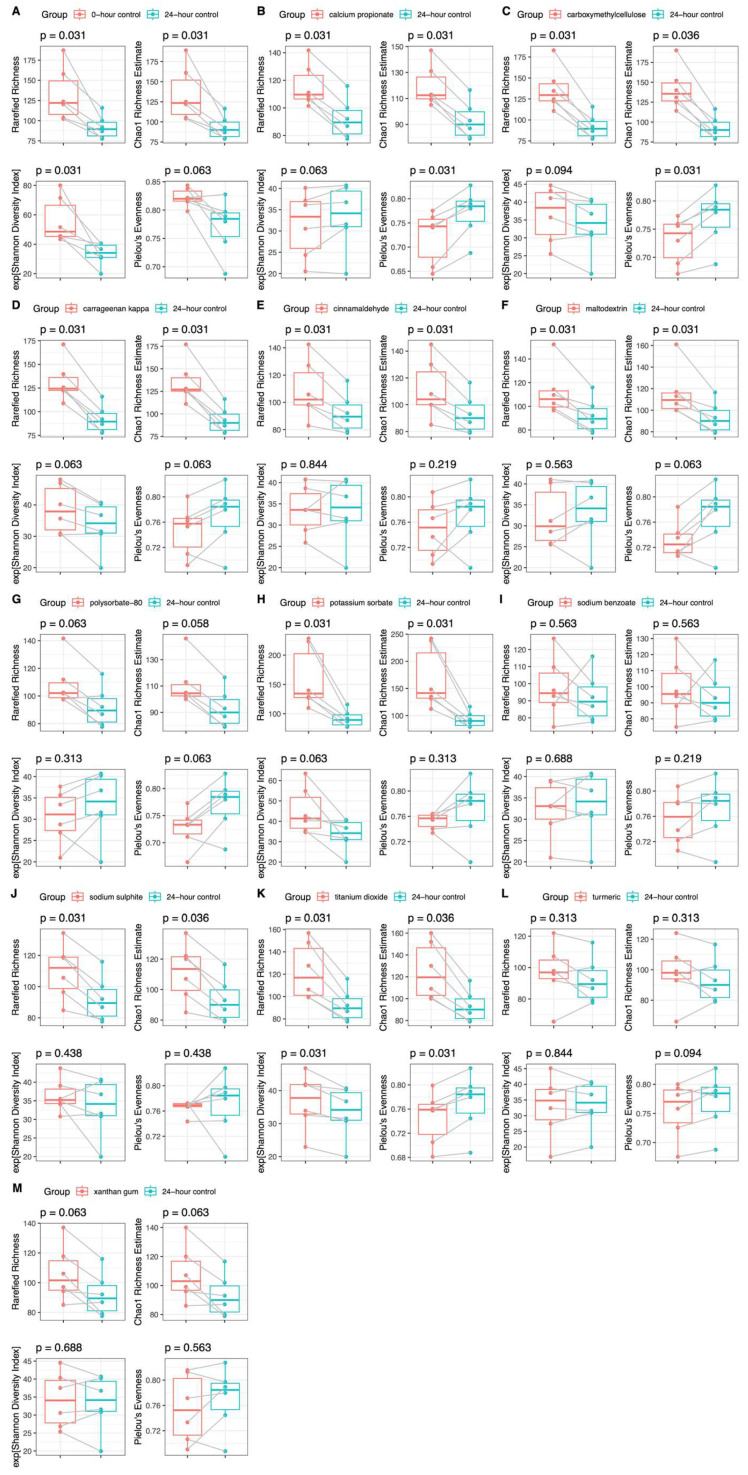
Microbiome α-diversity (Shannon diversity index, rarefied richness, Chao 1 richness estimate, and Pielou’s evenness) in HCs (*n* = 6) at baseline (**A**) and after 24 h batch faecal fermentation of fibre with various food additives (**B**–**M**) compared to the no food additive control.

**Figure 3:**
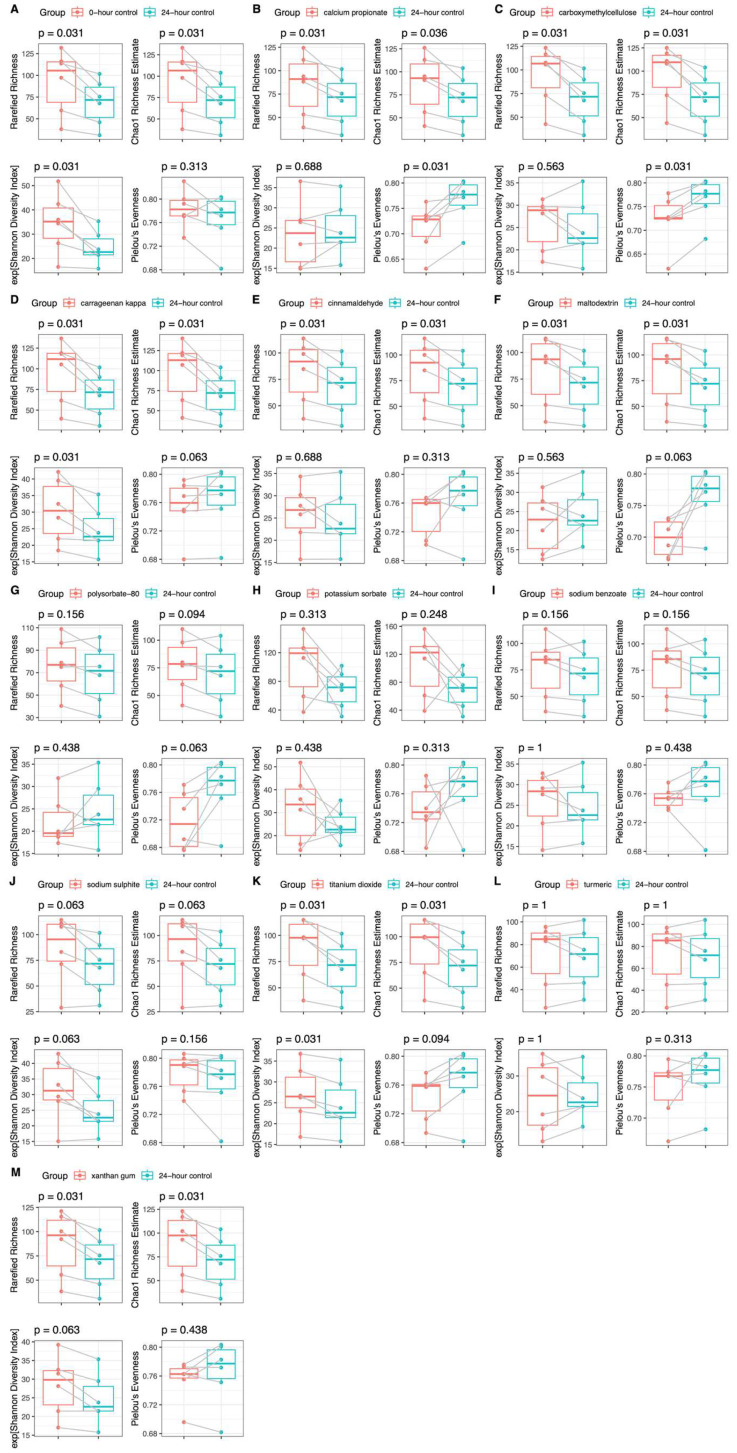
Microbiome α-diversity (Shannon diversity index, rarefied richness, Chao 1 richness estimate, and Pielou’s evenness) in patients with CD (*n* = 6) at baseline (**A**) and after 24 h batch faecal fermentation of fibre with various food additives (**B**–**M**) compared to the no food additive control.

Addition of titanium dioxide increased the metrics of microbiome α-diversity, including richness and the Shannon diversity index, in both HC and CD groups compared with the no-additive control (24hr-CNT) (Figure 2 and Figure 3). The Shannon diversity index also increased in the presence of carrageenan kappa in the CD group, but not in the HC group, although richness increased in both participant groups. In both groups, the addition of calcium propionate and carboxymethylcellulose increased richness but decreased evenness. Cinnamaldehyde and maltodextrin increased only richness in both groups (Figure 2 and Figure 3). Potassium sorbate and sodium sulphite increased richness in the HC group but not in the CD group, whereas xanthan gum increased richness in the CD group but not in the HC group. The remaining food additives did not significantly affect any metrics of microbiome α-diversity in either group.

### 3.3. Effect of Additives on Microbiome Community Structure

**Figure 4:**
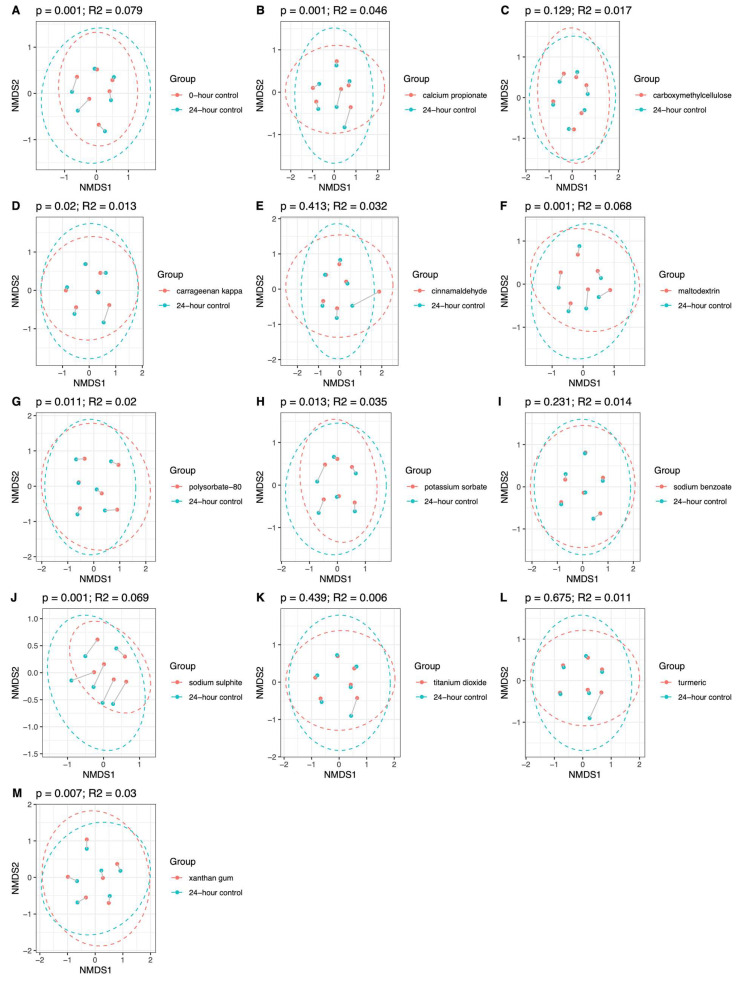
NMDS plot to show microbiome community structure β-diversity (Bray–Curtis dissimilarity index) in HCs (*n* = 6) at baseline (**A**) and after 24 h batch faecal fermentation of fibre with various food additives (**B**–**M**) compared to the no food additive control.

**Figure 5:**
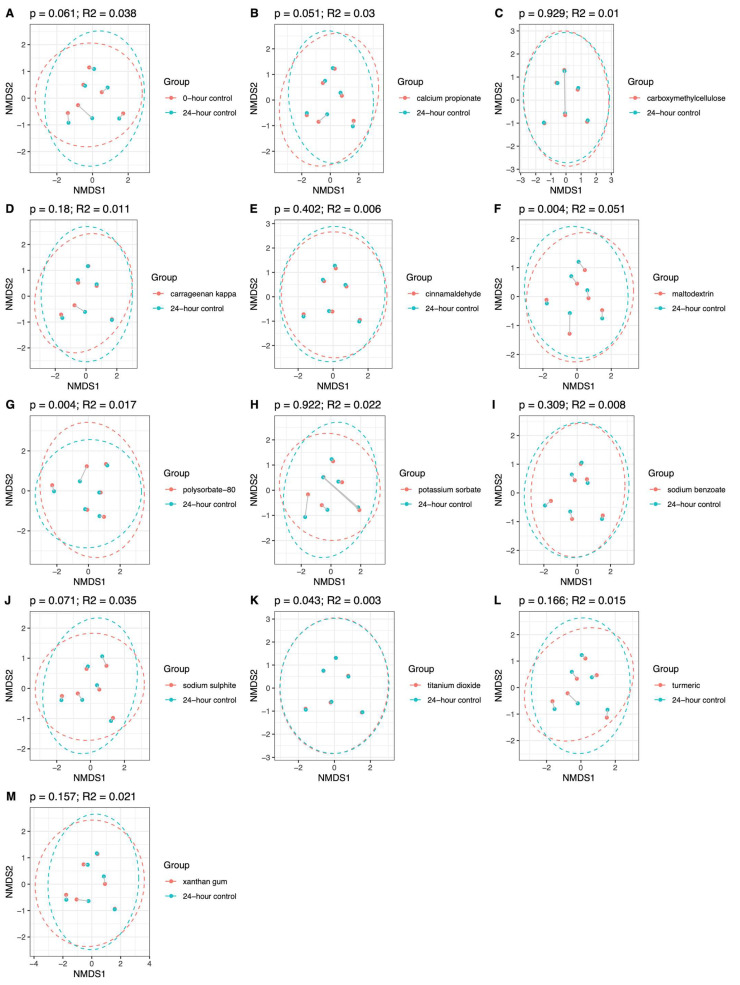
NMDS plots to show microbiome community structure β-diversity (Bray–Curtis dissimilarity index) in patients with CD (*n* = 6) at baseline (**A**) and after 24 h batch faecal fermentation of fibre with various food additives (**B**–**M**) compared to the no food additive control.

In both groups, the addition of maltodextrin and polysorbate-80 induced significant shifts in microbiome community structure (β-diversity), using the Bray–Curtis dissimilarity index (Figure 4 and Figure 5). In HCs, the most pronounced effects were observed for sodium sulphite, followed by maltodextrin, which explained 6.9% and 6.8% (*p* = 0.001) of the variance in microbiome community structure, respectively. In patients with CD, the most pronounced effect was from maltodextrin, explaining 5.1% (*p* = 0.004) of the variance in microbiome community structure.

The effects of the other food additives were significant but less pronounced. These included potassium sorbate (HC: R^2^ = 3.5%; *p* = 0.013), calcium propionate (HC: R^2^ = 4.6%; *p* = 0.001), polysorbate-80 (HC: R^2^ = 2%; *p* = 0.011; CD: R^2^ = 1.7%; *p* = 0.004), carrageenan kappa (HC: R^2^ = 1.3%; *p* = 0.02,), xanthan gum (HC: R^2^ = 3%: *p* = 0.007), and titanium dioxide (CD: R^2^ = 0.3%; *p* = 0.043).

### 3.4. Effect of Food Additives on Total Bacteria Taxon Absolute Abundance

**Figure 6:**
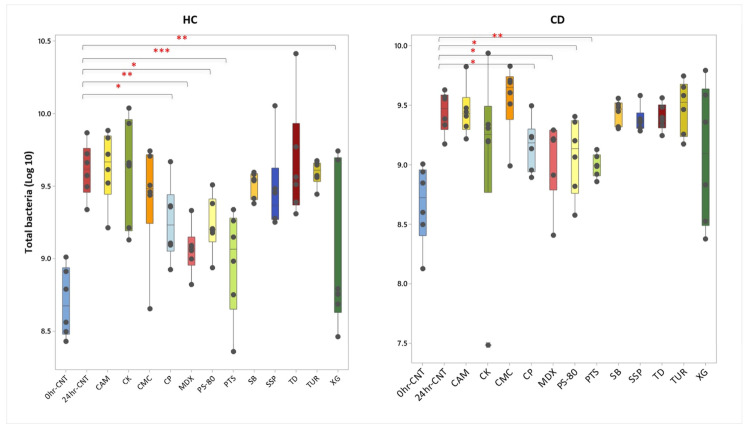
Concentration of total bacteria (number of 16S rRNA gene copies/mL ferment) before and following 24 h batch faecal fermentation of mixed fibre with different food additives in HCs (*n* = 6) and CD patients (*n* = 6). CP: calcium propionate; CMC: carboxymethylcellulose; CK: carrageenan kappa; CAM: cinnamaldehyde; MDX: maltodextrin; PS-80: polysorbate-80; PTS: potassium sorbate; SB: sodium benzoate; SSP: sodium sulphite; TD: titanium dioxide; TUR: turmeric; and XG: xanthan gum. Each dot represents an individual value (* = *p* < 0.05, ** = *p* < 0.01, *** = *p* < 0.001 compared to 24hr-CNT).

As expected, the concentration of total bacteria significantly increased after 24 h of fermentation in the no-additive controls for both participant groups, by an order of magnitude (Figure 6). Compared to the no-additive control, the addition of polysorbate-80 (*p* = 0.018; *p* = 0.025), potassium sorbate (*p* = 0.003; *p* < 0.0001), maltodextrin (*p* = 0.015; *p* = 0.001), and calcium propionate (*p* = 0.048; *p* = 0.033) decreased the concentration of total bacteria in both CD and HC groups, respectively. The addition of xanthan gum decreased the total bacteria concentration in the HC group (*p* = 0.001), but not in the CD group. Because of these effects, taxon relative abundances were expressed as estimated absolute values using the qPCR estimates of total bacterial load.

**Figure 7:**
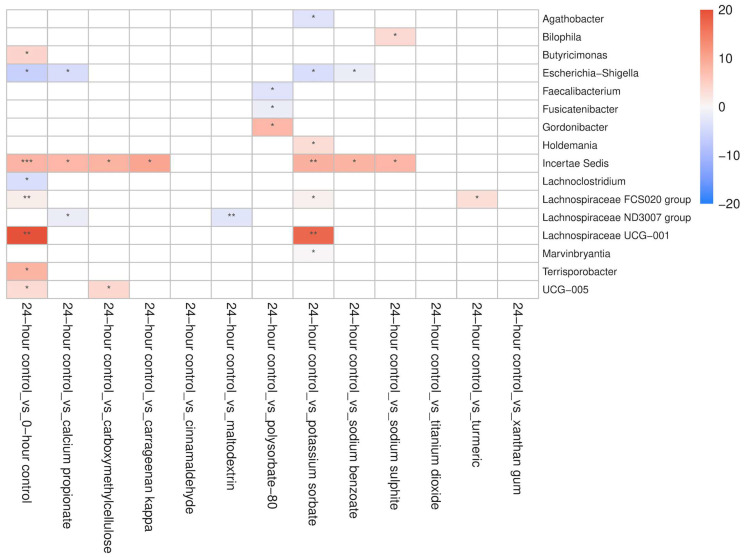
The effect of food additives (calcium propionate, carboxymethylcellulose, carrageenan kappa, cinnamaldehyde, maltodextrin, polysorbate-80, potassium sorbate, sodium benzoate, sodium sulphite, titanium dioxide, turmeric, and xanthan gum) on the estimated absolute abundance of bacterial genera in HCs (*n* = 6). Differences in abundance are expressed as log2 fold-difference between groups; red indicates increased abundance, while blue indicates decreased abundance (* = *p* < 0.05, ** = *p* < 0.01, *** = *p* < 0.001 compared to 24hr-CNT).

**Figure 8:**
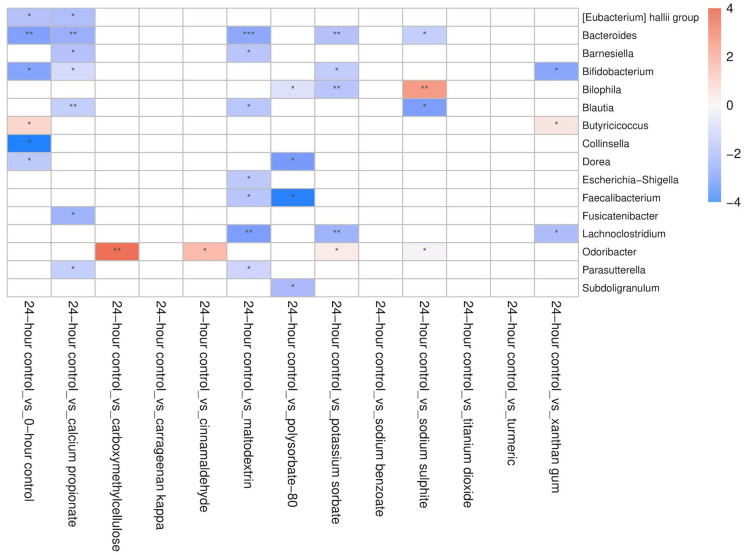
The effect of food additives (calcium propionate, carboxymethylcellulose, carrageenan kappa, cinnamaldehyde, maltodextrin, polysorbate-80, potassium sorbate, sodium benzoate, sodium sulphite, titanium dioxide, turmeric, and xanthan gum) on the absolute abundance of bacterial genera in CD patients (*n* = 6). Differences in abundance are expressed as log2 fold-difference between groups; red indicates increased abundance, while blue indicates decreased abundance (* = *p* < 0.05, ** = *p* < 0.01, *** = *p* < 0.001 compared to 24hr-CNT).

The addition of maltodextrin reduced the absolute abundance of *Escherichia–Shigella* in CD patients (*p* = 0.016), whereas sodium benzoate (*p* = 0.05), potassium sorbate (*p* = 0.038), and calcium propionate (*p* = 0.026) did so in HCs. The absolute abundance of *Bacteroides* also decreased with the addition of potassium sorbate (*p* = 0.008), calcium propionate (*p* = 0.005), sodium sulphite (*p* = 0.04), and maltodextrin (*p* < 0.005), but only in CD patients. Turmeric promoted the growth of *Lachnospiracea* in HCs (*p* = 0.036), while potassium sorbate (*p* = 0.033), calcium propionate (*p* = 0.034), and xanthan gum (*p* = 0.021) significantly inhibited the growth of *Bifidobacterium* in CD patients. Similarly, the absolute abundance of *Faecalibacterium* decreased in the presence of polysorbate-80 in HCs (*p* = 0.029), and in CD patients (*p* = 0.013), as well as with maltodextrin (*p* = 0.05) in CD patients. Compared to the no-additive control, potassium sorbate (*p* = 0.007) and polysorbate-80 (*p* = 0.039) significantly inhibited the growth of *Bilophila* in CD patients, whereas sodium sulphite promoted its growth in both HCs (*p* = 0.041) and CD patients (*p* = 0.039) (Figure 7 and Figure 8).

## 4. Discussion

This study investigated the impact of food additives on gut microbiome composition and SCFA production in patients with CD in remission and healthy individuals using an in vitro human faecal microbiome batch culture. Collectively, our findings show that the effects of each food additive on SCFA production and gut microbiome differ among individuals, yet with only modest differences according to the health status of participants. For example, maltodextrin increased acetate in both CD patients and HCs and reduced butyrate in HCs. In contrast, turmeric increased both acetate and butyrate only in HCs, whereas sodium sulphite reduced acetate specifically in HCs. Several additives altered α-diversity metrics, while maltodextrin and polysorbate-80 induced the strongest β-diversity changes in both groups, with sodium sulphite showing the most pronounced effect in HCs. Additives also modulated the absolute abundance of specific taxa by mainly inhibiting beneficial organisms such as *Bifidobacterium*, *Faecalibacterium* and *Bacteroides*, as well as potential pathobionts such as *Escherichia–Shigella* and *Bilophila.* Notably, sodium sulphite promoted *Bilophila* growth, whereas turmeric increased *Lachnospiraceae* in HCs.

Previous research has shown that certain food preservatives, such as sodium benzoate, which are widely used for their antimicrobial properties to prevent food spoilage, can alter the gut microbial community and exert pro-inflammatory effects. In mice, potassium sorbate increased the abundance of *Parabacteroides*, sodium nitrite increased *Akkermansia* and decreased *Erysipelotrichaceae*, and benzoic acid increased *Bacteroides* and *Ruminococcus* [26]. These alterations of the gut microbiome composition may compromise gut barrier function and increase intestinal epithelial permeability, which in turn may allow gut bacteria to interact more directly with the host’s immune system [12]. Likewise, in an animal study, sodium benzoate reduced levels of the probiotic *Bifidobacterium*, induced significant shifts in microbial community structure (β-diversity), and decreased overall microbial diversity (α-diversity) [11]. Notably, in a rodent model, a combination of antimicrobial food additives (sodium benzoate, sodium nitrite, and potassium sorbate) was found to induce gut microbial dysbiosis by inhibiting the growth of *Clostridiales* and promoting the growth of the *Proteobacteria* phylum [27]. In contrast, in a rat model of ulcerative colitis, sodium benzoate demonstrated anti-inflammatory and antioxidant effects, as indicated by reduced myeloperoxidase and increased glutathione levels [28]. In our present study, we observed an effect of sodium benzoate only in the HC group, but not in the CD group, where it decreased the absolute abundance of *Escherichia–Shigella*. Similarly, an in vitro study on healthy individuals reported that sodium benzoate promoted the growth of *Bifidobacterium* while inhibiting *E. coli* [15]. Such effects suggest that disruption of the gut microbiome by certain food additives may play a role in the underlying pathogenesis of IBD, which, if confirmed within clinical trials, may guide the development of dietary strategies for disease management.

Potassium sorbate is a widely used food preservative with good stability and high solubility, and it is commonly added to numerous products, such as bread, pastries, cereals, salad dressings, and sauces to preserve freshness, extend shelf-life, improve appearance and taste, and enhance overall quality [29]. Nevertheless, several studies have found that certain food preservatives may disrupt gut homeostasis and negatively affect gastrointestinal system function and immunity. For example, Peng et al. [30] reported that potassium sorbate exposure reduced the abundance of *Faecalibacterium* and altered the microbial community structure compared to the control group in zebrafish. *Faecalibacterium* is one of the most abundant members of the colonic microbiome in healthy subjects but is often found in low abundance in patients with CD [31]. It is a major butyrate producer with anti-inflammatory effects, such as blocking NF-κB activation and IL-8 secretion [32]. In line with these findings, the current study showed that potassium sorbate selectively decreased the absolute abundance of *Bifidobacterium* and *Bacteroides* in patients with CD, but not in HCs. These findings suggest that certain beneficial bacteria, such as *Faecalibacterium* and *Bifidobacterium*, may be negatively affected by certain food preservatives.

Calcium propionate is a widely used preservative that inhibits bacterial and fungal growth in bakery products, including bread. However, its effect on human health is not well-studied. In the current study, calcium propionate inhibited *Escherichia–Shigella* in HCs but also suppressed the growth of *Bifidobacterium*, *Eubacterium hallii* and *Blautia*, organisms with beneficial properties to the host, in patients with CD. *E. hallii* is known to contribute to host health by producing SCFAs, including butyrate; therefore, its depletion may negatively impact intestinal homeostasis [33]. Supporting these findings, Abd-Elhakim et al. [34] reported that mice exposed for 90 consecutive days to several food preservatives, including calcium propionate, potassium sorbate, sodium benzoate, and boric acid, showed elevated levels of inflammatory cytokines (IFNγ, TNF-α, IL-6, IL-1β, IL-10 and IL-4) compared to control littermates. This suggests that long-term exposure to food preservatives may trigger immune response and initiate inflammation.

Sodium sulphite is a widely used food preservative in alcoholic beverages and processed foods, where it prevents spoilage, discolouration, and oxidative reactions. Previous studies have shown that sodium sulphite strongly inhibits the growth of beneficial bacteria, including *F. prausnitzii* [35], *L. casei*, *L. plantarum*, *L. rhamnosus*, and *Streptococcus thermophilus* [36]. In the present study, sodium sulphite significantly increased the absolute abundance of the genus *Bilophila* in both HC and CD groups; it also induced a marked shift in microbial composition and decreased the production of acetate in HC. *Bilophila* species use sulphite for anaerobic respiration, producing hydrogen sulfide as a byproduct [10]. Elevated sulphite availability, therefore, provides a favourable environment for the growth of *B. wadsworthia.* Importantly, *B. wadsworthia* has been implicated in several diseases, including IBD and colorectal cancer [37]; its overgrowth may also exert systemic inflammatory effects by elevating pro-inflammatory cytokines, including IL-6 [38]. These findings suggest that food additives like sodium sulphite may induce microbial changes that promote inflammation, but replication in clinical trials is needed before any recommendations can be made.

Non-absorbed food additives that reach the colon might have a direct effect on the gut microbiome and the intestinal mucosa, particularly synthetic emulsifiers such as carboxymethylcellulose and polysorbate-80 [39]. Several animal and human studies have investigated the effect of emulsifier exposure on the gut microbiome [14,40,41]. For example, Rousta et al. [42] compared the effects of carboxymethylcellulose and polysorbate-80 on the human gut microbiome using a colitis model of ex-germ-free IL10-/- mice colonised with a pooled faecal transplant from three patients with active IBD. They found that carboxymethylcellulose increased lipocalin 2, a biomarker of intestinal inflammation, and elevated the expression of inflammatory cytokines, including TNF-α, compared to polysorbate-80 and water controls. Naimi et al. [39] used the MiniBioreactor Array Model to assess 20 common dietary emulsifiers ex vivo and showed that polysorbate-80 inhibited *F. prausnitzii* in patients with CD. Furthermore, Gerasimidis et al. [15] found that polysorbate-80 significantly decreased *Bifidobacterium* and the *Clostridium leptum* group, of which *F. prausnitzii* is a member, whereas carboxymethylcellulose did not significantly affect SCFA production or overall microbiome community structure. These results are consistent with our own findings; carboxymethylcellulose had no measurable impact on major SCFA production, while polysorbate-80 significantly reduced the absolute abundance of *Faecalibacterium* in both HC and CD groups and decreased *Subdoligranulum* in the CD group, while suppressing bacterial growth. Several studies have shown a reduced abundance of *Faecalibacterium* species in CD patients compared to healthy subjects [43,44]. Hence, one might hypothesise that certain emulsifiers may induce or exacerbate microbial alterations associated with CD pathogenesis. Jin et al. [45] showed that polysorbate-80 reduced the mucus layer by decreasing the mucin 2 RNA expression and increasing intestinal permeability. This suggests that chronic exposure to certain emulsifiers, such as polysorbate-80, may compromise the mucus layer of the gut and lead to increased adherence by pathogens, which may in turn contribute to inflammation. In contrast to the findings of the current study, an in vitro batch incubation model of the human gut microbiome using samples from ten healthy donors showed no changes in SCFA production after exposure to carboxymethylcellulose and polysorbate-80, which had minimal impact on the gut microbiome [46]. This points to the need for replication of preclinical studies within well-designed RCTs.

Xanthan gum is a commonly used thickener and stabiliser that can also contribute to emulsion stability. In our study, we found reduced growth of *Bifidobacterium* and *Lachnoclostridium* in patients with CD when xanthan gum was added, as well as alterations in microbial composition and decreased bacterial load in HCs. Similarly, Naimi et al. [39] reported that xanthan gum and carrageenans altered the microbiome composition and density using an ex vivo MiniBioreactor array model. Interestingly, a recent study by Katsoudas et al. [47] demonstrated that patients with IBD had the highest daily exposure to xanthan gum compared to HCs (0.39 ± 0.42 vs. 0.28 ± 0.42, *p* = 0.03). These results suggest that a higher dietary exposure to certain thickeners and emulsifiers in IBD patients may exacerbate microbial imbalance by suppressing the growth of beneficial bacterial taxa.

In contrast to the adverse effects on the gut microbiome observed for certain food additives, we observed potential favourable effects for some. For example, the addition of maltodextrin, a commonly used bulking agent and stabiliser, significantly reduced the absolute abundance of *Escherichia–Shigella* in the CD—taxa which have been implicated in promoting gut inflammation and dysbiosis in patients with IBD [19] and increased production of acetate, a precursor of butyrate, in both groups. It is possible that production of acetate reduced luminal pH, thus suppressing the growth of pathogenic bacteria, such as *E. coli* [48]. However, this in vitro model does not consider host immunity; therefore, it is difficult to determine whether these microbial changes are clinically beneficial or harmful to the host. A randomised, double-blind, placebo-controlled study reported increased *Bifidobacterium* growth in the placebo group that received 2.5 g of maltodextrin in powder form [49]. This result should be interpreted with caution, as such a low dosage of maltodextrin is unlikely to produce a profound effect. Gonza et al. [50] used a colonic in vitro fermentation model to investigate the impact of polysorbate-80, sucralose, titanium dioxide, sodium nitrite, and maltodextrin on the gut microbiome of five patients in remission, five with active IBD, and five healthy controls. Maltodextrin had a bifidogenic effect in all three groups, with the *Bifidobacterium* growth being higher in IBD than in healthy donors. It is important to note, however, that evidence on the impact of maltodextrin remains inconsistent. In an animal model, a diet supplemented with maltodextrin has been shown to lead to endoplasmic reticulum stress in intestinal goblets, which may cause mucus barrier dysfunction, thereby enhancing colitis susceptibility [51]. Additionally, feeding mice maltodextrin has led to decreased microbiome diversity, compositional changes, and reduced acetate levels [52]. The apparent inconsistency among findings from animal studies, in vitro fermentation models, and human trials may be explained by major methodological differences. In vitro fermentation models expose the gut microbiota directly to fixed concentrations of food additives, without accounting for digestion, absorption, host metabolism, or the immune system. Similarly, animal studies often use excessively high doses [40] and controlled feeding regimens, which can lead to detectable microbiome alterations and inflammatory outcomes. Indeed, recent feeding studies in humans observed less pronounced effects of food additives on gut microbiome [14,53] due to multiple confounding factors, such as dietary variability, differences in additive exposure level (as additives are consumed within multiple complex diets rather than as a single compound), and environmental influences. Therefore, this further increases the need for replication in well-controlled studies.

This study observed an increased growth of *Lachnospiraceae,* as well as elevated acetate and butyrate production when turmeric was added to the HC group. This is most likely because turmeric, a culinary spice and food additive, contains both curcumin and polysaccharides; it is the polysaccharides that bacteria can ferment using glucosyl hydrolase enzymes, which in turn may increase the production of SCFAs [54]. Nevertheless, these findings were not replicated in a previous double-blind, randomised, placebo-controlled pilot study [54], which found no significant effects on gut microbiome but did report an individualised response to the turmeric supplement.

Collectively, our findings suggest that while different food additives can influence gut microbiome composition and its fermentation capacity differently, we did not observe major differences between HCs and CD patients in remission. This may be explained by the gut microbiome of patients with IBD in remission having recovered sufficient functional stability to respond in a manner similar to that of healthy individuals when challenged with food additives. This aligns with data reported by Gerasimidis et al. [55], who found no differences in fibre fermentation capacity between HCs and patients with IBD in remission, despite significant differences in microbiome composition.

The present study has some limitations. While the in vitro batch culture 24 h fermentation with human inoculum is a valuable tool for short-term, well-controlled experiments free from host-related factors such as diet, inflammation and the local immune system, it does not precisely simulate effects in vivo. One of the limitations of this study is that the in vitro concentrations tested may not directly reflect oral intake as colonic luminal exposure is influenced by factors such as absorption, host metabolism and intestinal dilution, rather than the ingested amount. This information remains largely unknown for many food additives, which may be absorbed or further metabolised by the host. However, for some food additives such as carboxymethyl cellulose and carrageenans, which are not fermented by the gut microbiota, the majority of the orally ingested amount is expected to reach the colon. In addition, this model only reflects short-term effects of food additives and does not account for long-term adaptation of the gut microbiome. The small sample size of the two groups could have limited the statistical power of some findings. Furthermore, this study may be underpowered to detect subtle changes in taxon abundance. Future studies with larger sample sizes are needed to confirm the smaller microbial effects. All results were reported at *p* < 0.05, and multiple testing corrections were not applied to minimise false negative findings; however, this may have increased the risk of false positive results. As this is an exploratory study, the unadjusted values still provide a good basis for future investigation. Nevertheless, the use of an in vitro batch model in our study provided a clearer understanding of the comparative effect of each food additive on gut microbiome composition and fibre fermentation capacity in both healthy subjects and patients with CD in remission.

## 5. Conclusions

Certain food additives can affect SCFA production and alter the gut microbiome in distinct ways, with only modest differences observed according to the health status of participants. Future well-controlled clinical trials and mechanistic studies are needed to confirm these in vitro findings and to elucidate the role of food additives in health and disease, potentially informing the development of dietary strategies for disease management.

## Data Availability

Data may be available to other authors upon request.

## Supplementary Materials

The following supporting information can be downloaded at https://www.mdpi.com/article/10.3390/nu18040668/s1, Table S1: Functions and quantities of food additives added to each fermentation bottle; Figure S1: Net production of valerate, caproate, caprylate and enanthate and branched chain fatty acids (BCFAs) (iso-butyrate and iso-valerate) after 24 h batch faecal fermentation of fibre with 12 different food additives and no food additive control in HCs. Results are reported as μmol/mL faecal. (* = *p* < 0.05, ** = *p* < 0.01, *** = *p* < 0.001 compared to no food additive control); Figure S2: Net production of valerate, caproate, caprylate and enanthate and branched chain fatty acids (BCFAs) (iso-butyrate and iso-valerate) after 24 h batch faecal fermentation of fibre with 12 different food additives and no food additive control in participants with CD. Results are reported as μmol/mL faecal (* = *p* < 0.05, ** = *p* < 0.01, *** = *p* < 0.001 compared to no food additive control).

## References

[1] Wright E.K., Ding N.S., Niewiadomski O. (2018). Management of inflammatory bowel disease. Med. J. Aust..

[2] Ananthakrishnan A.N. (2015). Epidemiology and risk factors for IBD. Nat. Rev. Gastroenterol. Hepatol..

[3] Rajca S., Grondin V., Louis E., Vernier-Massouille G., Grimaud J.-C., Bouhnik Y., Laharie D., Dupas J.-L., Pillant H., Picon L. (2014). Alterations in the Intestinal Microbiome (Dysbiosis) as a Predictor of Relapse After Infliximab Withdrawal in Crohnʼs Disease. Inflamm. Bowel Dis..

[4] Cronin P., Joyce S.A., O’Toole P.W., O’Connor E.M. (2021). Dietary Fibre Modulates the Gut Microbiota. Nutrients.

[5] Den Besten G., Van Eunen K., Groen A.K., Venema K., Reijngoud D.-J., Bakker B.M. (2013). The role of short-chain fatty acids in the interplay between diet, gut microbiota, and host energy metabolism. J. Lipid Res..

[6] Canani R.B. (2011). Potential beneficial effects of butyrate in intestinal and extraintestinal diseases. World J. Gastroenterol..

[7] Parada-Venegas D., De La Fuente López M., Dubois-Camacho K., Landskron G., Blokzijl T., Molina H., Casanova M.C., Cui Y., Liu M., Da Costa De Pina A.M. (2025). Butyrate suppresses mucosal inflammation in inflammatory bowel disease primarily through HDAC3 inhibition in monocytes and macrophages. FEBS J..

[8] Takahashi K., Nishida A., Fujimoto T., Fujii M., Shioya M., Imaeda H., Inatomi O., Bamba S., Andoh A., Sugimoto M. (2016). Reduced Abundance of Butyrate-Producing Bacteria Species in the Fecal Microbial Community in Crohn’s Disease. Digestion.

[9] Hou J.K., Abraham B., El-Serag H. (2011). Dietary Intake and Risk of Developing Inflammatory Bowel Disease: A Systematic Review of the Literature. Am. J. Gastroenterol..

[10] Devkota S., Chang E.B. (2015). Interactions between Diet, Bile Acid Metabolism, Gut Microbiota, and Inflammatory Bowel Diseases. Dig. Dis..

[11] Liu C., Zhan S., Tian Z., Li N., Li T., Wu D., Zeng Z., Zhuang X. (2022). Food Additives Associated with Gut Microbiota Alterations in Inflammatory Bowel Disease: Friends or Enemies?. Nutrients.

[12] Raoul P., Cintoni M., Palombaro M., Basso L., Rinninella E., Gasbarrini A., Mele M.C. (2022). Food Additives, a Key Environmental Factor in the Development of IBD through Gut Dysbiosis. Microorganisms.

[13] Rinninella E., Cintoni M., Raoul P., Mora V., Gasbarrini A., Mele M.C. (2021). Impact of Food Additive Titanium Dioxide on Gut Microbiota Composition, Microbiota-Associated Functions, and Gut Barrier: A Systematic Review of In Vivo Animal Studies. Int. J. Environ. Res. Public Health.

[14] Chassaing B., Compher C., Bonhomme B., Liu Q., Tian Y., Walters W., Nessel L., Delaroque C., Hao F., Gershuni V. (2022). Randomized Controlled-Feeding Study of Dietary Emulsifier Carboxymethylcellulose Reveals Detrimental Impacts on the Gut Microbiota and Metabolome. Gastroenterology.

[15] Gerasimidis K., Bryden K., Chen X., Papachristou E., Verney A., Roig M., Hansen R., Nichols B., Papadopoulou R., Parrett A. (2020). The impact of food additives, artificial sweeteners and domestic hygiene products on the human gut microbiome and its fibre fermentation capacity. Eur. J. Nutr..

[16] De Souza Lopes A., Elisabete Costa Antunes A., Idelça Aires Machado K., Sartoratto A., Cristina Teixeira Duarte M. (2023). The impact of antimicrobial food additives and sweeteners on the growth and metabolite production of gut bacteria. Folia Microbiol..

[17] Pérez-Burillo S., Molino S., Navajas-Porras B., Valverde-Moya Á.J., Hinojosa-Nogueira D., López-Maldonado A., Pastoriza S., Rufián-Henares J.Á. (2021). An in vitro batch fermentation protocol for studying the contribution of food to gut microbiota composition and functionality. Nat. Protoc..

[18] Gibson R., Eriksen R., Chambers E., Gao H., Aresu M., Heard A., Chan Q., Elliott P., Frost G. (2019). Intakes and Food Sources of Dietary Fibre and Their Associations with Measures of Body Composition and Inflammation in UK Adults: Cross-Sectional Analysis of the Airwave Health Monitoring Study. Nutrients.

[19] Levine A., Sigall Boneh R., Wine E. (2018). Evolving role of diet in the pathogenesis and treatment of inflammatory bowel diseases. Gut.

[20] Gerasimidis K., Bertz M., Hanske L., Junick J., Biskou O., Aguilera M., Garrick V., Russell R.K., Blaut M., McGrogan P. (2014). Decline in Presumptively Protective Gut Bacterial Species and Metabolites Are Paradoxically Associated with Disease Improvement in Pediatric Crohn’s Disease During Enteral Nutrition. Inflamm. Bowel Dis..

[21] Svolos V., Hansen R., Nichols B., Quince C., Ijaz U.Z., Papadopoulou R.T., Edwards C.A., Watson D., Alghamdi A., Brejnrod A. (2019). Treatment of Active Crohn’s Disease With an Ordinary Food-based Diet That Replicates Exclusive Enteral Nutrition. Gastroenterology.

[22] Kozich J.J., Westcott S.L., Baxter N.T., Highlander S.K., Schloss P.D. (2013). Development of a Dual-Index Sequencing Strategy and Curation Pipeline for Analyzing Amplicon Sequence Data on the MiSeq Illumina Sequencing Platform. Appl. Environ. Microbiol..

[23] Callahan B.J., McMurdie P.J., Rosen M.J., Han A.W., Johnson A.J.A., Holmes S.P. (2016). DADA2: High-resolution sample inference from Illumina amplicon data. Nat. Methods.

[24] Wang Q., Garrity G.M., Tiedje J.M., Cole J.R. (2007). Naive Bayesian classifier for rapid assignment of rRNA sequences into the new bacterial taxonomy. Appl. Env. Microbiol..

[25] Quast C., Pruesse E., Yilmaz P., Gerken J., Schweer T., Yarza P., Peplies J., Glöckner F.O. (2013). The SILVA ribosomal RNA gene database project: Improved data processing and web-based tools. Nucleic Acids Res..

[26] Nagpal R., Indugu N., Singh P. (2021). Distinct Gut Microbiota Signatures in Mice Treated with Commonly Used Food Preservatives. Microorganisms.

[27] Hrncirova L., Machova V., Trckova E., Krejsek J., Hrncir T. (2019). Food Preservatives Induce Proteobacteria Dysbiosis in Human-Microbiota Associated Nod2-Deficient Mice. Microorganisms.

[28] Walia D., Kaur G., Jaggi A.S., Bali A. (2019). Exploring the therapeutic potential of sodium benzoate in acetic acid-induced ulcerative colitis in rats. J. Basic Clin. Physiol. Pharmacol..

[29] Xiao N., Ruan S., Mo Q., Zhao M., Liu T., Feng F. (2024). Effects of potassium sorbate on systemic inflammation and gut microbiota in normal mice: A comparison of continuous intake and washout period. Food Chem. Toxicol..

[30] Peng Q., Chang H., Wang R., You Z., Jiang S., Ma C., Huo D., Zhu X., Zhang J. (2019). Potassium sorbate suppresses intestinal microbial activity and triggers immune regulation in zebrafish (*Danio rerio*). Food Funct..

[31] Lopez-Siles M., Duncan S.H., Garcia-Gil L.J., Martinez-Medina M. (2017). *Faecalibacterium prausnitzii*: From microbiology to diagnostics and prognostics. ISME J..

[32] Sokol H., Pigneur B., Watterlot L., Lakhdari O., Bermúdez-Humarán L.G., Gratadoux J.-J., Blugeon S., Bridonneau C., Furet J.-P., Corthier G. (2008). *Faecalibacterium prausnitzii* is an anti-inflammatory commensal bacterium identified by gut microbiota analysis of Crohn disease patients. Proc. Natl. Acad. Sci. USA.

[33] Seegers J.F.M.L., Bui T.P.N., De Vos W.M., Mojgani N., Dadar M. (2021). Remarkable Metabolic Versatility of the Commensal Bacteria Eubacterium hallii and Intestinimonas butyriciproducens: Potential Next-Generation Therapeutic Microbes. Probiotic Bacteria and Postbiotic Metabolites: Role in Animal and Human Health.

[34] Abd-Elhakim Y.M., Hashem M.M.M., Abo-EL-Sooud K., Ali H.A., Anwar A., El-Metwally A.E., Mahmoud E.A., Moustafa G.G. (2020). Involvement of tumor necrosis factor-α, interferon gamma-γ, and interleukins 1β, 6, and 10 in immunosuppression due to long-term exposure to five common food preservatives in rats. Gene.

[35] Loayza J.J.J., Kang S., Schooth L., Teh J.J., De Klerk A., Noon E.K., Zhang J., Hu J., Hamilton A.L., Wilson-O’Brien A. (2023). Effect of food additives on key bacterial taxa and the mucosa-associated microbiota in Crohn’s disease. The ENIGMA study. Gut Microbes.

[36] Irwin S.V., Fisher P., Graham E., Malek A., Robidoux A. (2017). Sulfites inhibit the growth of four species of beneficial gut bacteria at concentrations regarded as safe for food. PLoS ONE.

[37] Davies J., Mayer M.J., Juge N., Narbad A., Sayavedra L. (2024). *Bacteroides thetaiotaomicron* enhances H_2_ S production in *Bilophila wadsworthia*. Gut Microbes.

[38] Feng Z., Long W., Hao B., Ding D., Ma X., Zhao L., Pang X. (2017). A human stool-derived *Bilophila wadsworthia* strain caused systemic inflammation in specific-pathogen-free mice. Gut Pathog..

[39] Naimi S., Viennois E., Gewirtz A.T., Chassaing B. (2021). Direct impact of commonly used dietary emulsifiers on human gut microbiota. Microbiome.

[40] Chassaing B., Koren O., Goodrich J.K., Poole A.C., Srinivasan S., Ley R.E., Gewirtz A.T. (2015). Dietary emulsifiers impact the mouse gut microbiota promoting colitis and metabolic syndrome. Nature.

[41] Furuhashi H., Higashiyama M., Okada Y., Kurihara C., Wada A., Horiuchi K., Hanawa Y., Mizoguchi A., Nishii S., Inaba K. (2020). Dietary emulsifier polysorbate-80-induced small-intestinal vulnerability to indomethacin-induced lesions via dysbiosis. J. Gastroenterol. Hepatol..

[42] Rousta E., Oka A., Liu B., Herzog J., Bhatt A.P., Wang J., Habibi Najafi M.B., Sartor R.B. (2021). The Emulsifier Carboxymethylcellulose Induces More Aggressive Colitis in Humanized Mice with Inflammatory Bowel Disease Microbiota Than Polysorbate-80. Nutrients.

[43] Fujimoto T., Imaeda H., Takahashi K., Kasumi E., Bamba S., Fujiyama Y., Andoh A. (2013). Decreased abundance of *F aecalibacterium prausnitzii* in the gut microbiota of C rohn’s disease. J. Gastroenterol. Hepatol..

[44] Cao Y., Shen J., Ran Z.H. (2014). Association between *Faecalibacterium prausnitzii* Reduction and Inflammatory Bowel Disease: A Meta-Analysis and Systematic Review of the Literature. Gastroenterol. Res. Pract..

[45] Jin G., Tang Q., Ma J., Liu X., Zhou B., Sun Y., Pang X., Guo Z., Xie R., Liu T. (2021). Maternal Emulsifier P80 Intake Induces Gut Dysbiosis in Offspring and Increases Their Susceptibility to Colitis in Adulthood. mSystems.

[46] Miclotte L., De Paepe K., Rymenans L., Callewaert C., Raes J., Rajkovic A., Van Camp J., Van De Wiele T. (2020). Dietary Emulsifiers Alter Composition and Activity of the Human Gut Microbiota in vitro, Irrespective of Chemical or Natural Emulsifier Origin. Front. Microbiol..

[47] Katsoudas N., Tavakoli P., Wu N., Shapiro A., Leach S.T., Williams A.-J., Paramsothy R., Ghaly S., Connor S.J., Samocha-Bonet D. (2024). Dietary Emulsifier Exposure in People With Inflammatory Bowel Disease Compared With Healthy Controls: Is There a Cause for Concern?. Inflamm. Bowel Dis..

[48] Pinhal S., Ropers D., Geiselmann J., De Jong H. (2019). Acetate Metabolism and the Inhibition of Bacterial Growth by Acetate. J. Bacteriol..

[49] Calgaro M., Pandolfo M., Salvetti E., Marotta A., Larini I., Pane M., Amoruso A., Del Casale A., Vitulo N., Fiorio M. (2021). Metabarcoding analysis of gut microbiota of healthy individuals reveals impact of probiotic and maltodextrin consumption. Benef. Microbes.

[50] Gonza I., Goya-Jorge E., Douny C., Boutaleb S., Taminiau B., Daube G., Scippo M.L., Louis E., Delcenserie V. (2024). Food additives impair gut microbiota from healthy individuals and IBD patients in a colonic in vitro fermentation model. Food Res. Int..

[51] Laudisi F., Stolfi C., Monteleone G. (2019). Impact of Food Additives on Gut Homeostasis. Nutrients.

[52] Zangara M.T., Ponti A.K., Miller N.D., Engelhart M.J., Ahern P.P., Sangwan N., McDonald C. (2022). Maltodextrin Consumption Impairs the Intestinal Mucus Barrier and Accelerates Colitis Through Direct Actions on the Epithelium. Front. Immunol..

[53] Wellens J., Vanderstappen J., Hoekx S., Vissers E., Luppens M., Van Elst L., Lenfant M., Raes J., Derrien M., Verstockt B. (2025). Effect of Five Dietary Emulsifiers on Inflammation, Permeability, and the Gut Microbiome: A Placebo-controlled Randomized Trial. Clin. Gastroenterol. Hepatol..

[54] Peterson C.T., Vaughn A.R., Sharma V., Chopra D., Mills P.J., Peterson S.N., Sivamani R.K. (2018). Effects of Turmeric and Curcumin Dietary Supplementation on Human Gut Microbiota: A Double-Blind, Randomized, Placebo-Controlled Pilot Study. J. Evid. -Based Integr. Med..

[55] Gerasimidis K., Nichols B., McGowan M., Svolos V., Papadopoulou R., Kokkorou M., Rebull M., Bello Gonzalez T., Hansen R., Russell R.K. (2022). The Effects of Commonly Consumed Dietary Fibres on the Gut Microbiome and Its Fibre Fermentative Capacity in Adults with Inflammatory Bowel Disease in Remission. Nutrients.

