# Supplementary material for: In Vitro Effects of Twelve Food Additives on Gut Microbiome and Its Fibre Fermentation Capacity in Adults with Crohn’s Disease in Remission and Healthy Controls
Source: Nutrients. 2026 Feb 18;18(4):668. doi: 10.3390/nu18040668 (PMC12942771; doi:10.3390/nu18040668)
Supplement: Supplementary file 1 [file nutrients-18-00668-s001.zip › nutrients-4146622-supplementary.pdf]

# Supplementary Materials

**Table S1. Functions and quantities of food additives added to each fermentation bottle.**

| Food Additive | Food Additive Function | Concentration Added to Each Bottle (mg/mL)-Liquid Food Additive Only | Volume/Mass Added to Each Fermentation Bottle | % Acceptable Daily Intake/ Estimated Daily Consumption Added to the Bottle |
|---|---|---|---|---|
| **Calcium propionate** | Preservative | | 281.25 mg | 50 |
| **Carrageenan-kappa** | Emulsifier | | 500 mg | 8.9 |
| **Carboxymethyl cellulose** | Emulsifier | | 500 mg | 27 |
| **Cinnamaldehyde** | Flavouring agent | 26 | 1 mL | 50 |
| **Turmeric** | Flavouring agent, colourant | | 250 mg | 40 |
| **Maltodextrin** | Thickener | | 500 mg | 0.83 |
| **Polysorbate-80** | Emulsifier | 500 | 1 mL | 27 |
| **Potassium sorbate** | Preservative | | 412.5 mg | 50 |
| **Sodium benzoate** | Preservative | | 187 mg | 50 |
| **Sodium sulphite** | Preservative | | 26 mg | 50 |
| **Titanium dioxide** | Colourant | | 75 mg | 50 |
| **Xanthan gum** | Thickener | | 500 mg | 4.29 |

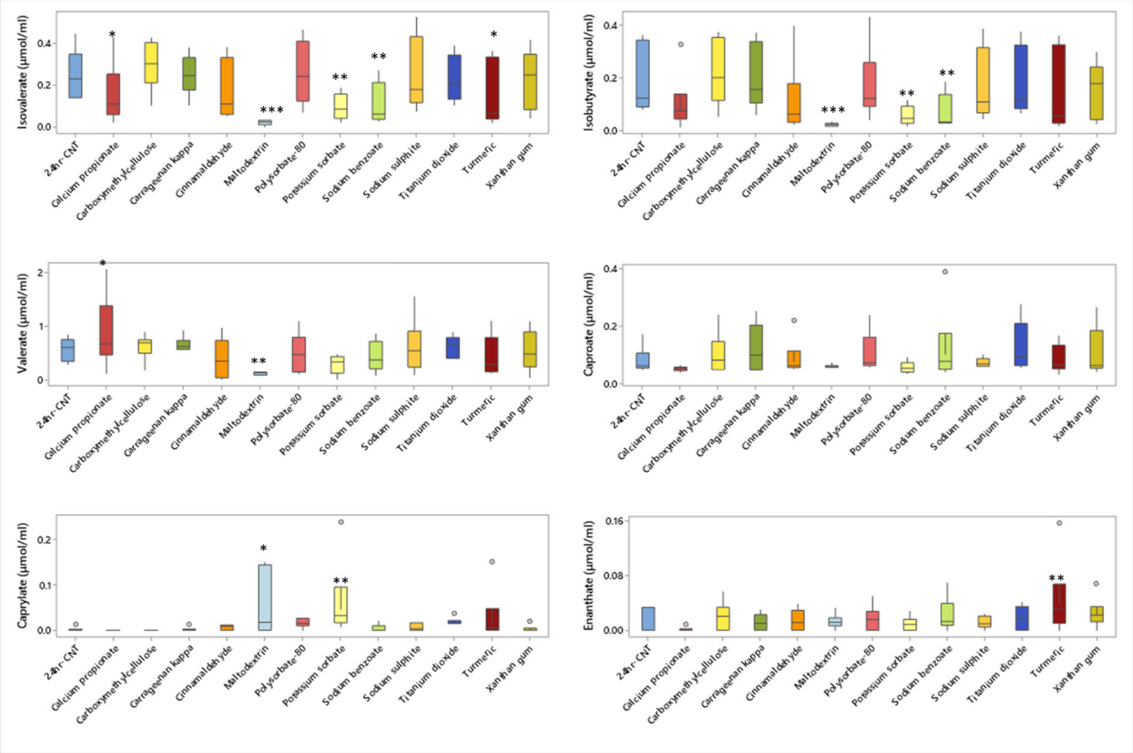


**Figure S1.** Net production of valerate, caproate, caprylate and enanthate and branched chain fatty acids (BCFA)(iso-butyrate and iso-valerate) after 24-hour batch faecal fermentation of fibre with 12 different food additives and no food additive control in participants with HC. Results are reported as μmol/ml faecal. (* $p < 0.05$, ** $p < 0.01$, ***$p < 0.001$ compared to no food additive control).

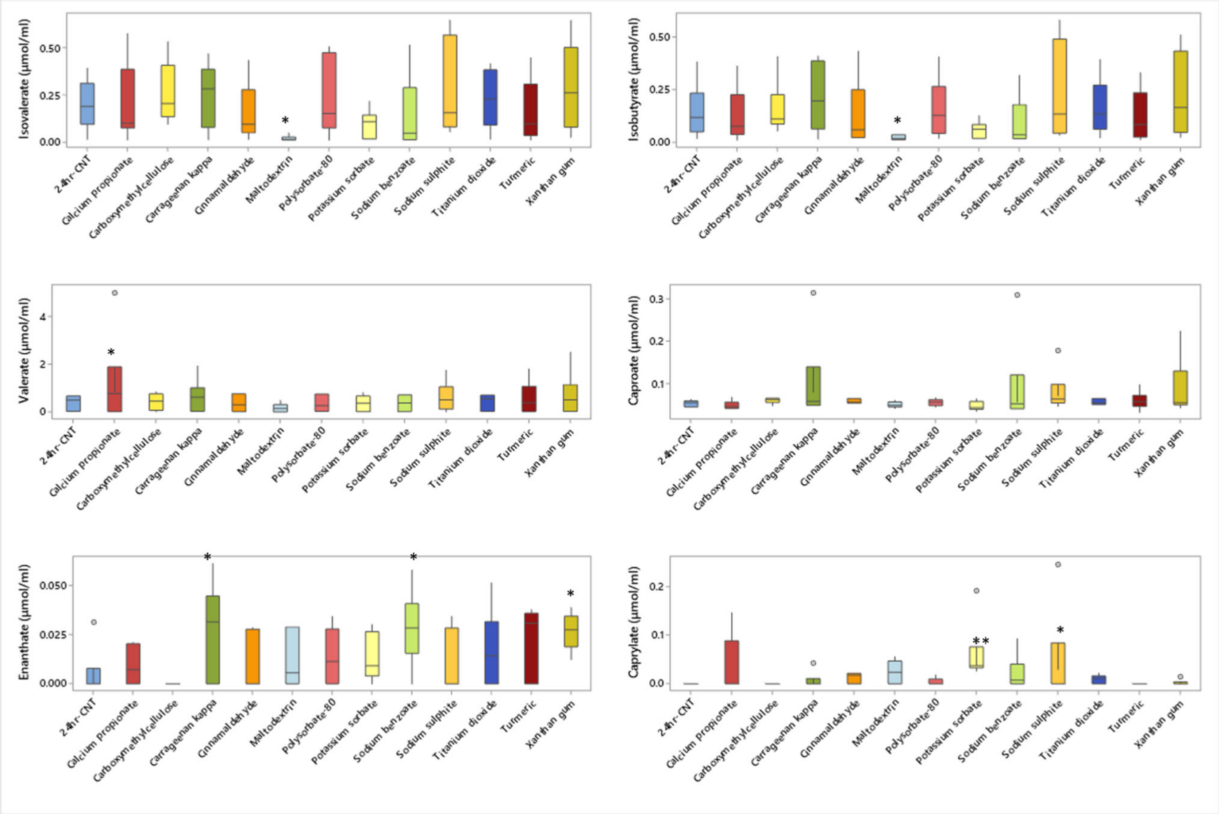


**Figure S2.** Net production of valerate, caproate, caprylate and enanthate and branched chain fatty acids (BCFA)(iso-butyrate and iso-valerate) after 24-hour batch faecal fermentation of fibre with 12 different food additives and no food additive control in participants with CD. Results are reported as µmol/ml faecal. (* $p < 0.05$, ** $p < 0.01$, *** $p < 0.001$ compared to no food additive control).
